# Response to inhaled granulocyte‐macrophage colony‐stimulating factor in patient with mild‐to‐moderate autoimmune pulmonary alveolar proteinosis—24 months of follow‐up

**DOI:** 10.1111/crj.13650

**Published:** 2023-06-10

**Authors:** Iván Oterino‐Moreira, María‐Jesús Linares‐Asensio, Sira Sanz‐Márquez, Montserrat Pérez‐Encinas

**Affiliations:** ^1^ Pharmacy Department Alcorcon Foundation University Hospital Alcorcon Spain; ^2^ Pulmonology Department Alcorcon Foundation University Hospital Alcorcon Spain

**Keywords:** aerosol drug therapy, granulocyte‐macrophage colony‐stimulating factor, inhalation drug administration, pulmonary alveolar proteinosis, sargramostim

## Abstract

**Introduction:**

Autoimmune pulmonary alveolar proteinosis (aPAP) is a disease caused by IgG antibodies against granulocyte‐macrophage colony‐stimulating factor (GM‐CSF). Whole lung lavage (WLL) allows to remove the lipo‐proteinaceous material accumulated by the poor clearance of alveolar surfactant. However, it is a complex technique that is not exempt from complications, and in some cases, the patients are refractory, requiring the performance of several WLLs spaced apart in time.

**Materials and Methods:**

We present the clinical, functional, and radiological evolution after 24 months of follow‐up of a patient diagnosis of aPAP refractory to WLL, with performed three therapeutic WLLs spaced 16 and 36 months and serious potentially fatal complications in the last one.

**Results and Disscusion:**

After 24 months, no adverse effects have appeared and the great clinical, functional and radiological response is maintained. The patient has been successfully treated with inhaled recombinant human GM‐CSF sargramostim.

## Graphical abstract

After failure of three whole lung lavages (WLLs) in less than 5 years, the administration of sargramostim allowed the patient to avoid WLL that he had been performing, with only temporary improvement and risk of serious associated complications. After 2 years of follow‐up, no adverse effects have been associated with the experimental therapy, and the response has been excellent.

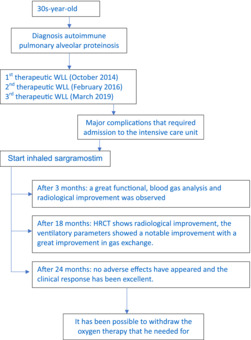

## 1 INTRODUCTION

Pulmonary alveolar proteinosis (PAP) is a rare disease characterized by the accumulation in the pulmonary alveoli of lipoprotein material derived from pulmonary surfactant, causing alterations in gas exchange.^1^ The pathogenesis is related to decreased surfactant clearance due to alterations in the phagocytic capacity of alveolar macrophages.^1^ Three main categories of PAP have been defined depending on the etiology: autoimmune (primary or idiopathic), secondary, and genetic.^2^

The treatment of choice is whole lung lavage (WLL).^2^ This method removes lipo‐proteinaceous material accumulated in alveoli surface by lavage to improve breath function with response rate 70%–84%.^3^

Classically, the procedure has been performed under a general anesthesia with rigid bronchoscope, after selective intubation and mechanical ventilation.^2^ Some centers used flexible fiberoptic bronchoscope (FFB) instead of selective intubation under a general anesthesia and mechanical ventilation. Yet, selective intubation and mechanical ventilation may be the cause of severe complications such as barotrauma, pneumothorax, or infections.^2^, ^3^

In addition, Froudarakis et al. have reported successful and uncomplicated WLL performed by FFB in a 13‐year‐old girl awake under local anesthesia only, avoiding general anesthesia, selective intubation, and mechanical ventilation.^4^ This procedure is generally safe yet less effective because of the use of a small lavage volume, so it is only indicated when a patient cannot tolerate general anesthesia or presents with less severe disease or in pediatric cases.^2^

Autoimmune pulmonary alveolar proteinosis (aPAP) accounts for 90% of all PAP cases and is caused by IgG antibodies against GM‐CSF, which are necessary for the final differentiation and maturation of the alveolar macrophage to occur.^1^ Thus, adding exogenous GM‐CSF, by subcutaneous injection or inhalation, is an alternative for the treatment of aPAP.^3^

There are currently no randomized clinical trials investigating the use of inhaled recombinant human GM‐CSF in aPAP,^5^ so evidence is supported by some observational studies and meta‐analyses.^3^, ^6^, ^7^, ^8^ However, the duration of treatment in the literature is variable, and an optimal guideline has not yet been established.

We present the clinical, radiological, and functional evolution of a case successfully treated with inhaled GM‐CSF, during 24 months of treatment.

## 2 MATERIALS AND METHODS

A late 30s‐year‐old male with smoking history (21 package year) and no other risk factors of interest presents in April 2014 FVC 3570 mL (69%), FEV1 2630 mL (63%), RV 55%, TLCO53%, and KCO 69% with persistent pulmonary infiltrates suggestive of alveolar proteinosis. Clinically, the patient has moderate restrictive ventilatory impairment, moderate diffusion impairment, and moderate–severe hypoxemic respiratory failure with desaturation in the walk test that requires a chronic home oxygen therapy program. Anti‐GM‐CSF antibodies in serum were analyzed with a positive result: 24.6 U/mL [<5 U/mL], confirming the diagnosis of aPAP.

In the following 5 years, given the functional worsening, the patient required three therapeutic WLLs spaced 16 and 36 months apart (October 2014, February 2016, and March 2019).

In the last therapeutic WLL, the patient developed major complications that required admission to the intensive care unit (acute respiratory distress syndrome, with severe bronchospasm, need for extracorporeal membrane oxygenation, encephalopathy in the context of severe desaturation, and bilateral pulmonary thromboembolism due to thrombosis of the right jugular vein).

**FIGURE 1:**
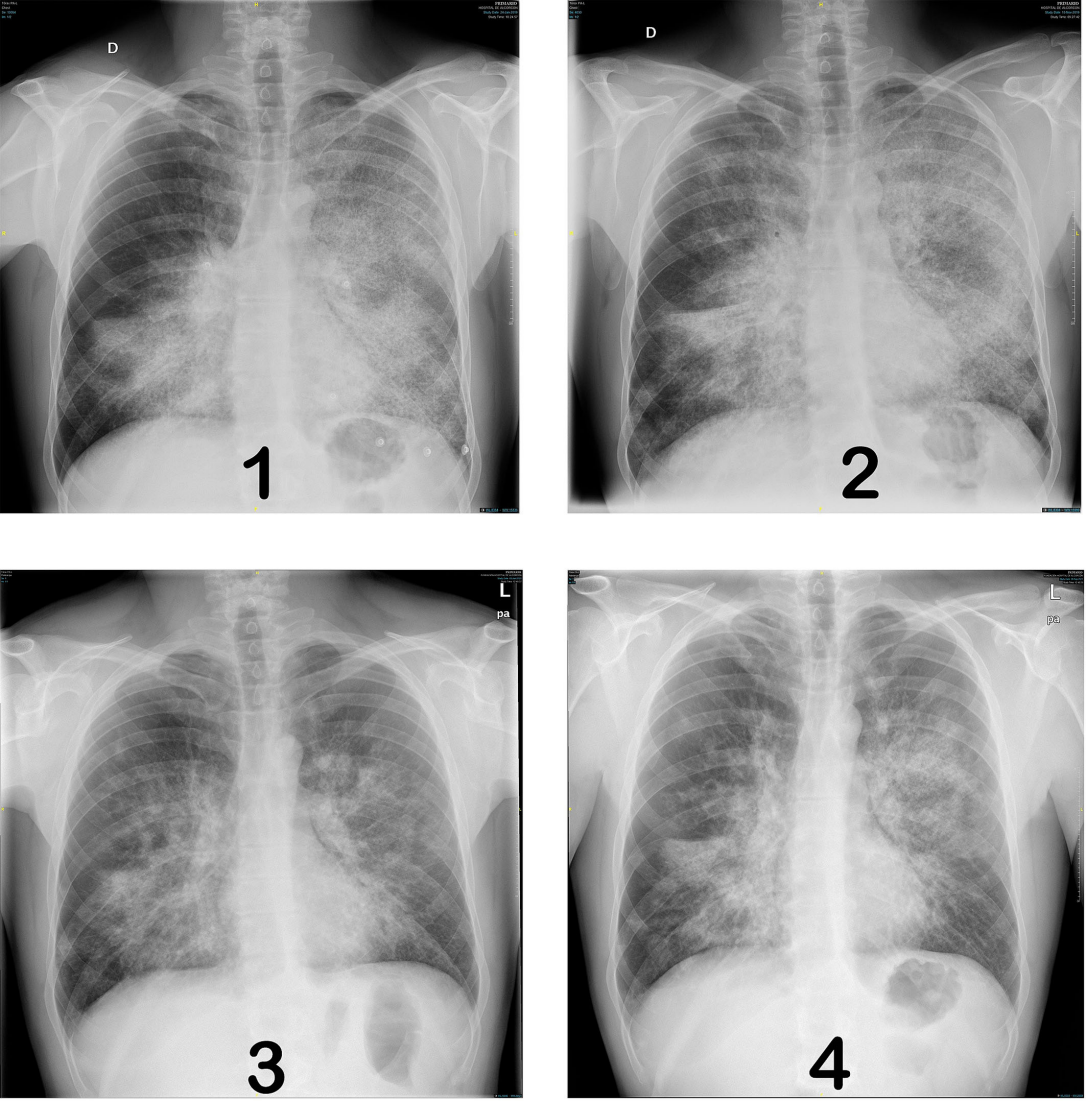
Radiological evolution (chest X‐ray). Situation before third WLL,^1^ further worsening after 7 months after third WLL,^2^ improvement after 3 months of treatment with inhaled GM‐CSF,^3^ and stability after 18 months of treatment.^4^

In October 2019 (7 months after third WLL), he presented again a rapid decline in clinical, functional, and radiological evolution (see Table 1 and Figure 1), so off‐label treatment with recombinant human sargramostim GM‐CSF was chosen.

**TABLE 1:** Clinical and functional evolution of the ventilatory parameters and the 6‐min walk test.

	3 months after third WLL	7 months after third WLL	3 months of treatment	6 months of treatment	18 months of treatment	24 months of treatment
**Pulmonary function test**						
FVC mL (%)	4000 (77)	3570 (69)	4240 (84)	4510 (90)	4330 (87)	4310 (87)
FFEV1 mL (%)	3110 (74)	2630 (63)	2690 (72)	3080 (75)	2900 (71)	3030 (75)
RV (%)	128	55	138	150	125	150
TLCO (%)	50	37	53	52	74	68
KCO (%)	65	59	69	64	85	74
**6MWT**						
Theoretical distance (m)	*	*	614.52	435.71	627.06	619.17
HR minute 6 (beats per minute)	*	*	136	108	108	105
O_2_ sat. minute 6 (%)	*	*	89	89	93	91

Abbreviations: bmp, beats per minute; FEV1, forced expiratory volume in 1 s; FVC, forced vital capacity; HR, heart rate; KCO, transfer coefficient of the lung for carbon monoxide; m, meters; O_2_ sat, oxygen saturation; RV, residual volume; TLCO, transfer factor of the lung for carbon monoxide.

250 μg of drug was administered every 12 hours for 7 days every other week, diluted in 5 mL of saline and inhaled through a mouthpiece coupled to a pneumatic nebulizer according to the recommendations of the Spanish Society of Pneumology and Thoracic Surgery,^9^ prior inhalation of salbutamol to prevent bronchospasm.

The authors have the written informed consent of the patient for access to their medical history and publication of the results.

## 3 RESULTS

After 3 months of treatment, a great functional, blood gas analysis and radiological improvement was observed. The chest radiograph showed radiological improvement of the bilateral alveolar pattern, with greater aeration of the entire left hemithorax and slight clearance of the infiltrate of the right hemithorax (Figure 1). FVC increased 21.7%, FEV1 14.3%, TLCO 43.2%, and KCO 16.9% (Table 1).

After 18 months, the comparison of the high‐resolution CT (HRCT) of the chest with the baseline before treatment shows radiological improvement with clearance of bilateral parenchymal infiltrates, and the chest X‐ray shows stability compared to the previous one (1 year post‐treatment) as can be seen in Figure 1. On the other hand, the ventilatory parameters showed a notable improvement with a great improvement in gas exchange. In addition, for the first time, the patient did not desaturate during the 6‐min walk test (Table 1).

After 24 months, no adverse effects have appeared, and the clinical response has been excellent, presenting normal total lung capacity and forced vital capacity (102% and 87%, respectively) with mild–moderate alteration of pulmonary diffusion (KCO in the limit lower than normal with slightly lower TLCO), without significant desaturation in the walk test (O_2_ Sat. = 96%), radiological stability in HRCT chest, and it has been possible to withdraw the oxygen therapy that he needed for ambulation.

## 4 DISCUSSION

We present a case treated with inhaled GM‐CSF after three whole lung lavages, with major complications after the last one. The treatment has generated great satisfaction for the patient with excellent clinical, functional and radiological response with significant improvement in gas exchange, which has allowed home oxygen therapy to be withdrawn.

The pharmacy department supported off‐label treatment by means of a favorable report to the regulatory agency. Also, in the absence of package leaflet, the pharmacy department prepared individualized information for the patient about the drug and treatment.

In the case presented by Rodríguez Portal et al., a 32‐year‐old woman who started therapy with inhaled GM‐CSF after two whole lung lavages, no adverse effects appeared after 12 months of treatment, and dyspnea went from grade 3 to 1 on the Medical Research Council scale with improvement in FVC from 37% to 51% and in DLCO from 23% to 51%, with withdrawal of home oxygen therapy.^6^ Gajewska et al. also reported a case of a 14‐year‐old boy treated with inhaled GM‐CSF after three whole lung lavages with clinical improvement after 17 months of treatment: FVC went from 70% to 79% and DLCO from 54% to 74%, with no adverse effects.^8^

As an alternative to inhaled GM‐CSF therapy, after failure or contraindication of whole lung lavage, other treatments such as subcutaneous GM‐CSF 250 mcg/day have been tried with similar response rates; however, unlike inhaled therapy, some mild adverse effects such as fever, fatigue, headache, and injection site complications are included. Other studies have also tested the use of the anti‐CD20 monoclonal antibody rituximab; however, it was associated with risks, and some of them serious.^1^, ^10^

After 2 years of follow‐up, no adverse effects have been associated with the experimental therapy, and it has allowed the patient to avoid the therapeutic WLL he had been undergoing, with only a temporary improvement and risk of associated serious complications.

In conclusion, our case supports that inhaled GM‐CSF has been safe and effective in the treatment of aPAP and represents a therapeutic option after resistance or contraindication to therapeutics whole lung lavage.

## AUTHOR CONTRIBUTIONS

Iván Oterino‐Moreira designed study, performed study, collected data, analyzed data, and wrote the paper. María‐Jesús Linares‐Asensio, Sira Sanz‐Márquez, and Montserrat Pérez‐Encinas analyzed data and contributed important reagents.

## CONFLICT OF INTEREST STATEMENT

The authors declare they have no conflicts of interest.

## ETHICS STATEMENT

The authors have the written informed consent of the patient for access to their medical history and publication of the results.

## Data Availability

The data that support the findings of this study are available on request from the corresponding author. The data are not publicly available due to privacy or ethical restrictions.

## References

[1] Rodríguez Portal JA . Treatment of adult primary alveolar proteinosis. Arch Bronconeumol. 2015;51(7):344‐349. doi:10.1016/j.arbres.2015.02.003 25896950

[2] Gay P , Wallaert B , Nowak S , et al. Efficacy of whole‐lung lavage in pulmonary alveolar proteinosis: a multicenter international study of GELF. J Thorac Dis. 2021;13(6):3539‐3548. doi:10.21037/jtd-20-3308 34277049 PMC8264701

[3] Zhen G , Li D , Jiang J , Weng Y . Granulocyte‐macrophage colony‐stimulating factor inhalation therapy for severe pulmonary alveolar Proteinosis. Am J Ther. 2020;28(2):e171‐e178. doi:10.1097/MJT.0000000000001053 31513019

[4] Froudarakis ME , Koutsopoulos A , Mihailidou HP . Total lung lavage by awake flexible fiberoptic bronchoscope in a 13‐year‐old girl with pulmonary alveolar proteinosis. Respir Med. 2007;101(2):366‐369. doi:10.1016/j.rmed.2006.04.020 16806874

[5] International Clinical Trials Registry Platform (ICTRP) Search Portal [Internet] . [accessed Mar 2023]. Available at: https://trialsearch.who.int/2

[6] Rodríguez Portal JA , Rodríguez Becerra E , Sánchez GA . Response to inhaled granulocyte‐macrophage colony‐stimulating factor in a patient with alveolar proteinosis. Arch Bronconeumol. 2009;45(3):150‐152. doi:10.1016/j.arbres.2008.02.006 19286115

[7] Sheng G , Chen P , Wei Y , Chu J , Cao X , Zhang H‐L . Better approach for autoimmune pulmonary alveolar proteinosis treatment: inhaled or subcutaneous granulocyte‐macrophage colony‐stimulating factor: a meta‐analysis. Respir Res. 2018;19(1):163. doi:10.1186/s12931-018-0862-4 30165864 PMC6117963

[8] Gajewska ME , Sritharan SS , Santoni‐Rugiu E , Bendstrup EM . Autoimmune pulmonary alveolar proteinosis in an adolescent successfully treated with inhaled rhGMCSF (molgramostim). Respir Med Case Rep. 2018;23:167‐169. doi:10.1016/j.rmcr.2018.02.005 29719809 PMC5925949

[9] Giner J , Basualdo LV , Casan P , et al. Guideline for the use of inhaled drugs. The Nursing Area of the Spanish Society of Pneumology and Thoracic Surgery. Arch Bronconeumol. 2000;36(1):34‐43. doi:10.1016/S0300-2896(15)30231-3 10726183

[10] Chan E , King T . Treatment and prognosis of pulmonary alveolar proteinosis in adults. UpToDate Aug 2021 [updated Jul 2022, accessed Mar 2023]. Available at: https://www.uptodate.com/

